# Machine learning and cancer survival: analysis of the Registro Hospitalar de Câncer do Estado de São Paulo

**DOI:** 10.11606/s1518-8787.2026060007389

**Published:** 2026-07-24

**Authors:** Fernando Henrique de Albuquerque Maia, Lucas Buk Cardoso, Simone Aldrey Angelo, Yasmin Pacheco Gil Bonilha, Adeylson Guimarães Ribeiro, Maria Paula Curado, Gisele Aparecida Fernandes, Alexandre Dias Porto Chiavegatto, Vanderlei Cunha Parro, Tatiana Natasha Toporcov

**Affiliations:** I Universidade de São Paulo. Faculdade de Saúde Pública. Departamento de Epidemiologia. São Paulo, SP, Brasil; II Instituto Mauá de Tecnologia. Núcleo de Sistemas Eletrônicos Embarcados. São Caetano do Sul, SP, Brasil; III Fundação Oncocentro de São Paulo. São Paulo, SP, Brasil; IV A.C. Camargo Cancer Center. Grupo de Epidemiologia e Estatística em Câncer. São Paulo, SP, Brasil

**Keywords:** Neoplasms, Survival Analysis, Machine Learning

## Abstract

**OBJECTIVE:**

To compare the performance of different Survival Machine Learning (SML) algorithms in predicting the survival of cancer patients.

**METHODS:**

Data from the Registro Hospitalar de Câncer do Estado de São Paulo (São Paulo State Cancer Registry Hospital) were used, covering the five most incident types of cancer (breast, prostate, lung, colorectal and cervix). Six algorithms were evaluated: Gradient Boosting Survival (GBS), Random Survival Forest (RSF), Support Vector Machine Survival (SVM-Survival), XGBoost Cox, XGBoost Accelerated Failure Time (AFT), and LightGBM. Performance was measured by the Concordance Index (C-Index), C-Index IPCW and Integrated Brier Score (IBS) metrics.

**RESULTS:**

The XGBoost AFT model showed the best C-Index results for breast (0.7845), lung (0.7368), colorectal (0.7618), and cervix (0.7726), while GBS was superior for prostate (0.7574). Clinical staging was consistently the most important variable, according to the explainability analysis.

**CONCLUSION:**

The SML algorithms showed good predictive performance, regardless of cancer type, sample size and censoring proportion. These models show potential for subsidizing cancer planning and supporting strategic decisions in the organization of cancer care networks.

## INTRODUCTION

The incidence of cancer has risen sharply in recent years due to an ageing population and changes in lifestyle habits, with an estimated 20 million new cases and 9.7 million deaths per year^1^. It is a major public health problem, with an impact on health systems around the world.

Survival analysis is a type of analysis in which the dependent variable of interest is the time until a certain event occurs^2^. In cancer, these studies aim to analyze the time between diagnosis and/or admission to a specialized service to treat the disease and the patient’s death^3^. An important aspect of this type of analysis is the inclusion of the observation time of censored individuals. Censoring occurs when a patient is lost to follow-up or when the study ends and they continue without having presented the outcome of interest. In contrast, analyses of dichotomous outcomes, such as five-year survival, need to use only those individuals for whom there is certainty about survival or death during the follow-up period, and it is necessary to exclude censored individuals from the analysis.

Survival analysis models based on traditional statistics depend on assumptions about the distribution of the data. The Cox proportional hazards model (Cox-PH) is the most widely used and is a semi-parametric model that assumes the proportionality of risks for different individuals is constant over time^3^. As such, its use requires tests to confirm that the assumptions are met, requiring adaptations and adjustments when this is not the case^2^. This model is often used to estimate the effect of explanatory variables on the risk of an event over time but can be adapted for individual risk prediction using estimators such as Breslow.

The use of Survival Machine Learning (SML) algorithms for survival analysis has been growing in recent years^4,5^. Their main advantage is the ability to analyze large data sets with variables of different formats, without the need to meet certain assumptions. This is especially useful in the case of attributes with non-linear associations with the variable of interest.

In Brazil, some studies have looked at cancer mortality and survival^6,7^, using data from the Registro Hospitalar de Câncer do Estado de São Paulo (RHC/SP – São Paulo State Cancer Registry Hospital) of the Fundação Oncocentro do Estado de São Paulo (FOSP – São Paulo State Oncocentro Foundation). However, these studies did not consider survival time as the dependent variable of interest, but rather the occurrence or non-occurrence of the event (death) after certain time intervals. Thus, in these analyses, it was necessary to exclude individuals due to loss to follow-up.

In a previous study, SML models were compared with machine learning models for dichotomous survival outcomes at one, three and five years in patients with colorectal cancer, showing that the dichotomous models underestimated the survival of these patients^8^. This article included patients with the five most frequent types of cancer in the state of São Paulo, which show significant differences in their incidence and in the proportion of censored cases. The main SML models were tested to identify the algorithms with the best prediction performance and possible differences in performance related to sample size and the proportion of censored cases.

## METHODS

The RHC/SP, managed by FOSP, gathers socioeconomic and clinical information on patients diagnosed with cancer since 2000, provided by 81 institutions in the state of São Paulo. This database, with anonymized information, is public and can be consulted by completing a registration form on the institution’s website^9^. For this study, cases of the five most common types of cancer in the state of São Paulo were selected: breast, prostate, lung, colorectal and cervix.

Before carrying out the study, the authors identified the variables that would be included in the model and the inclusion and exclusion criteria, considering the characteristics of the tumors and the available data set. The decision was made to exclude variables that showed high collinearity with the chosen variables, a low proportion of completeness or that were related to the type of treatment the individual had undergone. The intention was to work with data that is available before treatment begins, so that these models can be used in later studies to build scenarios and/or optimize the organization of the health services network.

The data sets for the five types of cancer under study were prepared in two stages: general selections and column adjustments – applied to all the topographies analyzed – and specific selections, made according to the particularities of each type. For all types of cancer, patients were excluded if they were under 20 years of age, did not live in the state of São Paulo, had an undefined clinical stage or had not been diagnosed, had carcinoma *in situ,* had no microscopic confirmation of the diagnosis, had uncertain morphology, or had undergone a bone marrow transplant.

In the specific selections, additional criteria were applied for each type of cancer. Patients with lung cancer who had received hormone therapy were excluded, since this treatment is generally intended for patients with other types of tumors with lung metastases. In the case of colorectal cancer, only patients with adenocarcinoma morphology (code 81403) were considered, since the other morphologies are rarer and show different behavior. For breast cancer, only women were included, given that this type of cancer in men is rare and behaves very differently.

The set of variables analyzed included individual patient information, such as age (IDADE), sex (SEXO), and schooling (ESCOLARI); variables related to place of residence, such as the city’s IBGE code (IBGE) and Regional Health Department (DRS); and variables relating to the care institution, including institution code (INSTITU), category of care (CATEATEND), IBGE code of the institution’s city (IBGEATEN), DRS of the institution (DRS_INST), and category of qualification in high complexity oncology (HABILIT2). Clinical variables were also considered, such as diagnosis prior to admission (DIAGPREV), topography (TOPO), morphology (MORFO), clinical staging (EC), and year of diagnosis (ANODIAG). Finally, the categorized time between the first consultation and the start of treatment (TRATCONS_CAT) and between diagnosis and the start of treatment (DIAGTRAT_CAT) were evaluated. The gender variable was only used for lung and colorectal cancer, the topography variable was not considered for prostate, and the morphology variable was not considered for colorectal. The data was divided into two parts, with 80% of the patients being used to train the survival algorithms and the other 20% to validate these models.

The outcome was the time between diagnosis and death from all causes, including censored patients. Six machine learning models were used for survival analysis: Gradient Boosting Survival (GBS), Random Survival Forest (RSF), Survival Support Vector Machine (SSVM), XGBoost Cox (XGB-Cox), XGBoost Accelerated Failure Time (XGB-AFT), and LightGBM (LGBM). Each adopts a different strategy for modeling the time until an event occurs and was chosen for their ability to deal with censorship in the data, capture non-linear patterns and provide more flexible predictions compared to traditional statistical approaches. The methodological aspects relating to the treatment of the database, as well as the specificities of each type of SML model, are described in more depth in another paper^8^.

Three metrics were used to evaluate the survival models: Concordance Index (C-Index), Inverse Probability of Censoring Weighted Concordance Index (C-Index IPCW), and Integrated Brier Score (IBS). The C-Index is a metric used to measure the discriminatory capacity of the model, i.e. its ability to correctly order the survival times of individuals^10^. Values close to 1 indicate perfect agreement, while a value of 0.5 suggests performance equivalent to chance. The C-Index IPCW is a variation of the C-Index that corrects for biases introduced by non-informative or covariate-dependent censoring^11^. Finally, the IBS measures the accuracy of predictions of survival probability over time and is calculated from the survival curves estimated by the models. Lower values indicate better performance, with 0 being a perfect prediction^12^. As IBS depends on the survival curves, it was only calculated for the models that had adequate survival curves and were consistent with the empirical observations.

The hyperparameters of the machine learning models were selected using Optuna^13^, using three different samplers. RandomSampler combines the parameters randomly; TPESampler^14^ optimizes the choice using probabilistic models; and CmaEsSampler^15^ employs an evolutionary strategy that adjusts a Gaussian distribution over the space of hyperparameters. Each sampler was evaluated with 150 different combinations, applying cross-validation in 10 folds for each set of parameters. For each type of cancer, the model with the best performance among the three samplers was selected.

The SHapley Additive exPlanations (SHAP^)^ and the Permutation Importance (PI)^17^ were used to assess the impact of the different variables on the predictions of the survival models. SHAP estimates the average contribution of each variable to the predictions, considering all possible combinations of values, while PI evaluates the loss of model performance when the values of a variable are randomly shuffled.

### Ethical Aspects

This study was carried out using a public database, anonymized and without sensitive variables that could identify individuals, preserving confidentiality. As such, it was not submitted to a Research Ethics Committee, in accordance with Resolution No. 510/2016 of the National Health Council.

## RESULTS

The database was obtained from the RHC/SP in September 2024 with data on 1,223,973 individuals. After general and specific selections by type of cancer, 141,726 patients with breast cancer, 111,406 with prostate cancer, 45,719 with lung cancer, 44,856 with colorectal cancer, and 27,850 with cervical cancer were included, as described in Chart 1.

**Chart 1:** Adjustments and selections made to the RHC/SP datasets.

	Total number of patients in RHC/SP	General exclusions	Specific selections	Total patients included
Breast cancer (C50)	170,413	Age < 20 years Residing in other states Clinical staging undefined or not performed No microscopic confirmation of diagnosis Uncertain morphology Carcinoma *in situ* Bone marrow transplant performed	Female	141,726
Prostate cancer (C61)	128,575		(No additional specific selection)	111,406
Lung cancer (C34)	57,620		No treatment with hormone therapy	45,719
Colorectal cancer (C18, C19, C20)	90,625		Adenocarcinoma (morphology = 81403)	44,856
Cervical cancer (C53)	56,912		(No additional specific selection)	27,850

RHC/SP: Hospital Cancer Registry of the state of São Paulo.

The profile of the patients included in the study is shown in Table 1. The number of patients included for each type of cancer ranged from 27,850 cervical cases to 141,726 breast cases, with 44,856 colorectal, 45,719 lung, and 111,406 prostate cases. In lung cancer and colorectal cancer, there was a predominance of male patients, corresponding to 60.2% and 52.0% of cases, respectively.

**Table 1:** Profile of patients included in the study.

	Breast n (%)	Prostate n (%)	Lungs n (%)	Colorectal n (%)	Cervix n (%)
Schooling					
Illiterate	4,997 (3.53)	5,257 (4.72)	2,418 (5.29)	1,670 (3.72)	2,066 (7.42)
Incomplete primary education	33,601 (23.71)	34,875 (31.30)	13,717 (30.00)	11,549 (25.75)	8,167 (29.33)
Complete primary education	22,536 (15.90)	18,557 (16.66)	8,967 (19.61)	8,198 (18.28)	4,527 (16.26)
High school	25,771 (18.18)	13,009 (11.68)	5,387 (11.78)	6,302 (14.05)	4,022 (14.44)
Higher education	15,651 (11.04)	7,986 (7.17)	3,022 (6.61)	3,431 (7.65)	1,197 (4.30)
No information	39,170 (27.64)	31,722 (28.47)	12,208 (26.70)	13,706 (30.56)	7,871 (28.26)
Age - Average (Median)	56.46 (56)	67.58 (68)	64.01 (64)	62.39 (63)	51.53 (51)
Sex					
Male	0 (0)	111,406 (100)	27,530 (60.21)	23,312 (51.97)	0 (0)
Female	141,726 (100)	0 (0)	18,189 (39.78)	21,544 (48.03)	27,850 (100)
Category of service					
Insurance	16,487 (11.63)	10,621 (9.53)	3,544 (7.75)	3,991 (8.90)	1,064 (3.82)
SUS	82,721 (58.37)	68,698 (61.66)	25,477 (55.72)	28,149 (62.75)	16,426 (58.98)
Private	906 (0.64)	1,691 (1.52)	341 (0.74)	299 (0.67)	74 (0.27)
No information	41,612 (29.36)	30,396 (27.28)	16,357 (35.77)	12,417 (27.68)	10,286 (36.93)
Previous diagnosis					
No previous diagnosis	69,411 (48.98)	36,225 (32.52)	29,816 (65.22)	18,255 (40.70)	11,761 (42.23)
With previous diagnosis	72,315 (51.02)	75,181 (67.48)	15,903 (34.78)	26,601 (59.30)	16,089 (57.77)
Topography	C500: 4,512 (3.18) C501: 6,651 (4.69) C502: 9,188 (6.48) C503: 5,195 (3.67) C504: 34,244 (24.16) C505: 6,339 (4.47) C506: 1,300 (0.92) C508: 21,803 (15.38) C509: 52,494 (37.04)	C619: 111,406 (100)	C340: 2,499 (5.47) C341: 12,982 (28.40) C342: 1,340 (2.93) C343: 6,736 (14.73) C348: 1,679 (3.67) C349: 20,483 (44.80)	C180: 1,371 (3.06) C181: 81 (0.18) C182: 3,547 (7.91) C183: 379 (0.84) C184: 1,616 (3.60) C185: 327 (0.73) C186: 1,996 (4.45) C187: 6,638 (14.80) C188: 297 (0.66) C189: 6,955 (15.51) C199: 2,791 (6.22) C209: 18,858 (42.04)	C530: 759 (2.73) C531: 1,243 (4.46) C538: 443 (1.59) C539: 25,405 (91.22)
Clinical staging					
I	33,780 (23.83)	9,133 (8.20)	4,936 (10.80)	5,378 (11.99)	8,346 (29.97)
II	54,970 (38.79)	65,986 (59.23)	2,937 (6.42)	12,400 (27.64)	6,544 (23.50)
III	38,224 (26.97)	18,659 (16.75)	12,037 (26.33)	14,393 (32.09)	8,722 (31.32)
IV	14,752 (10.41)	17,628 (15.82)	25,809 (56.45)	12,685 (28.28)	4,238 (15.22)
Categorized time between first consultation and start of treatment					
Up to 60 days	84,201 (59.41)	51,357 (46.10)	25,728 (56.27)	31,905 (71.13)	15,504 (55.67)
Between 61 and 90 days	23,604 (16.65)	13,299 (11.94)	5,056 (11.06)	5,030 (11.21)	4,709 (16.91)
More than 91 days	31,390 (22.15)	39,839 (35.76)	7,008 (15.33)	5,743 (12.80)	6,245 (22.42)
Not treated	2,531 (1.79)	6,911 (6.20)	7,927 (17.34)	2,178 (4.86)	1,392 (5.00)
Categorized time between diagnosis and start of treatment					
Up to 60 days	69,639 (49.14)	31,301 (28.10)	26,809 (58.64)	25,554 (56.97)	11,608 (41.68)
Between 61 and 90 days	26,803 (18.91)	14,725 (13.22)	5,035 (11.01)	7,019 (15.65)	5,211 (18.71)
More than 91 days	42,753 (30.17)	58,469 (52.48)	5,948 (13.01)	10,105 (22.53)	9,639 (34.61)
Not treated	2,531 (1.79)	6,911 (6.20)	7,927 (17.34)	2,178 (4.86)	1,392 (5.00)
Death from all causes					
No	95,069 (67.08)	75,027 (67.35)	6,808 (14.89)	22,979 (51.23)	15,333 (55.06)
Yes	46,657 (32.92)	36,379 (32.65)	38,911 (85.11)	21,877 (48.77)	12,517 (44.94)
Model split					
Training	113,380 (80.00)	89,124 (80.00)	36,575 (80.00)	35,884 (80.00)	22,280 (80.00)
Test	28,346 (20.00)	22,282 (20.00)	9,144 (20.00)	8,972 (20.00)	5,570 (20.00)
Total	141,726 (100)	111,406 (100)	45,719 (100)	44,856 (100)	27,850 (100)

The proportion of deaths from all causes varied considerably between the types of cancer included. Lung cancer cases had the highest proportion of deaths (85.1%), while breast and prostate had the lowest proportion of deaths, with values close to each other (32.9% and 32.7%, respectively). Colorectal (48.8%) and cervix (44.9%) were at intermediate levels. The distribution according to topography, as well as the division between training and testing can be found in the Supplementary Material^a^. The Kaplan-Meier curves stratified by clinical stage can also be found in the Supplementary Material.

Table 2 shows the performance of the SML models for predicting survival after optimizing the hyperparameters for the five types of cancer. In breast cancer, the XGB-AFT model showed the best performance in terms of C-Index (0.7845) and C-Index IPCW (0.7570), while GBS obtained the lowest IBS (0.1325). As for prostate cancer, GBS stood out with the best C-Index (0.7574) and C-Index IPCW (0.7332) values, as well as the lowest IBS (0.1298). In the lung cancer scenario, the XGB-AFT model obtained the highest C-Index (0.7368) and the highest IPCW C-Index (0.7325), although the lowest IBS was recorded by the GBS model (0.1165). For colorectal cancer, the XGB-AFT again showed the highest C-Index (0.7618) and C-Index IPCW (0.7532) values, while the lowest IBS was recorded by the GBS (0.1552). Finally, in the case of cervical cancer, the XGB-Cox model achieved the highest C-Index (0.7726), while the GBS obtained the highest IPCW C-Index (0.7643) and the lowest IBS (0.1473). The survival curves are presented in the Supplementary Material.

**Table 2:** Summary of the results obtained by the best models after searching for hyperparameters.

Models	Breast cancer			Prostate cancer			Lung cancer			Colorectal cancer			Cervical cancer		
	C-Index	C-Index IPCW	IBS	C-Index	C-Index IPCW	IBS	C-Index	C-Index IPCW	IBS	C-Index	C-Index IPCW	IBS	C-Index	IPCW C-Index	IBS
RSF	0.7784	0.7525	0.1335	0.7557	0.7300	0.1303	0.7247	0.7212	0.1192	0.7547	0.7489	0.1565	0.7664	0.7583	0.1481
GBS	0.7821	0.7565	**0.1324**	**0.7574**	**0.7332**	**0.1298**	0.7324	0.7288	**0.1165**	0.7588	0.7526	**0.1552**	0.7722	**0.7643**	**0.1473**
SSVM	0.7574	0.7301	0.1415	0.7094	0.6907	0.1424	0.6686	0.6669	0.1310	0.7088	0.7036	0.1774	0.7372	0.7315	0.1630
XGB - Cox	0.7021	0.6896	-	0.6747	0.6645	-	0.7212	0.7174	-	0.7240	0.7184	-	0.7452	0.7375	-
XGB - AFT	**0.7845**	**0.7570**	-	0.7574	0.7328	-	**0.7368**	**0.7325**	-	**0.7618**	**0.7532**	-	**0.7726**	0.7639	-
LGBM	0.7133	0.7000	-	0.6862	0.6742	-	0.7210	0.7170	-	0.7275	0.7211	-	0.7483	0.7404	-

Note: models that achieved the best performance for each tumor type and evaluated metric are highlighted in bold.

C-Index: Concordance Index; C-Index IPCW: Inverse Probability of Censoring Weighted Concordance Index; GBS: Gradient Boosting Survival; IBS: Integrated Brier Score; LGBM: LightGBM; RSF: Random Survival Forest; SSVM: Survival Support Vector Machine; XGB-Cox: XGBoost Cox; XGB-AFT: XGBoost Accelerated Failure Time.

Chart 2 shows the most relevant variables for predicting survival, identified using the SHAP and PI methods, for each type of cancer analyzed. The models selected were those with the best performance in Table 2 according to the IPCW C-Index: XGB-AFT for breast, lung and colorectal; GBS for prostate and cervix. The variable CE (clinical staging) was consistently classified as the most important in the breast, prostate, cervical and colorectal cancer models, by both the SHAP and PI methods, while in lung cancer it was the most important only according to the SHAP method, and in the PI method the categorized time between consultation and treatment was the most important variable. The Figure shows the distribution of SHAP values.

**Chart 2:** Importance of features according to the SHAP and Permutation Importance methods.

	Breast cancer		Prostate cancer		Lung cancer		Colorectal cancer		Cervical cancer	
	XGB - AFT		GBS		XGB - AFT		XGB - AFT		GBS	
	SHAP	PI	SHAP	PI	SHAP	PI	SHAP	PI	SHAP	PI
1	EC	EC	EC	EC	EC	TRATCONS_CAT	EC	EC	EC	EC
2	IDADE	IDADE	IDADE	IDADE	TRATCONS_CAT	EC	IDADE	TRATCONS_CAT	DIAGTRAT_CAT	CATEATEND
3	CATEATEND	CATEATEND	CATEATEND	CATEATEND	DIAGTRAT_CAT	DIATRAT_CAT	CATEATEND	IDADE	CATEATEND	DIAGTRAT_CAT
4	ANODIAG	ANODIAG	DIAGTRAT_CAT	DIAGTRAT_CAT	MORFO	MORFO	TRATCONS_CAT	INSTITU	IDADE	IDADE
5	IBGEATEN	IBGEATEN	ANODIAG	ANODIAG	TOPO	INSTITU	DIAGPREV	CATEATEND	ANODIAG	ANODIAG
6	DIAGTRAT_CAT	INSTITU	DIAGPREV	INSTITU	SEXO	TOPO	INSTITU	ANODIAG	TRATCONS_CAT	INSTITU
7	TOPO	TRATCONS_CAT	TRATCONS_CAT	TRATCONS_CAT	DIAGPREV	DIAGPREV	DIAGTRAT_CAT	DIAGTRAT_CAT	IBGEATEN	TRATCONS_CAT
8	DIAGPREV	ESCOLARI	INSTITU	IBGEATEN	IDADE	IDADE	ANODIAG	TOPO	INSTITU	IBGEATEN
9	ESCOLARI	DIAGTRAT_CAT	MORFO	DRS_INST	CATEATEND	CATEATEND	TOPO	IBGEATEN	HABILIT2	ESCOLARI
10	TRATCONS_CAT	TOPO	ESCOLARI	HABILIT2	INSTITU	ANODIAG	SEXO	DIAGPREV	ESCOLARI	MORFO

ANODIAG: year of diagnosis; CATEATEND: category of care; DIAGPREV: diagnosis prior to admission; DIAGTRAT_CAT: categorized time between diagnosis and start of treatment; DRS: Regional Health Department; DRS_INST: Regional Health Department of the institution; EC: clinical staging; ESCOLARI: schooling; GBS: Gradient Boosting Survival; HABILIT2: high complexity oncology qualification category; IBGE: IBGE code of the city of residence; IBGEATEN: IBGE code of the city of the institution; IDADE: age; INSTITU: institution code; MORFO: tumor morphology; PI: Permutation Importance; SHAP: SHapley Additive exPlanations; TOPO: tumor topography; TRATCONS_CAT: categorized time between first consultation and start of treatment; XGB-AFT: XGBoost Accelerated Failure Time.

**Figure:**
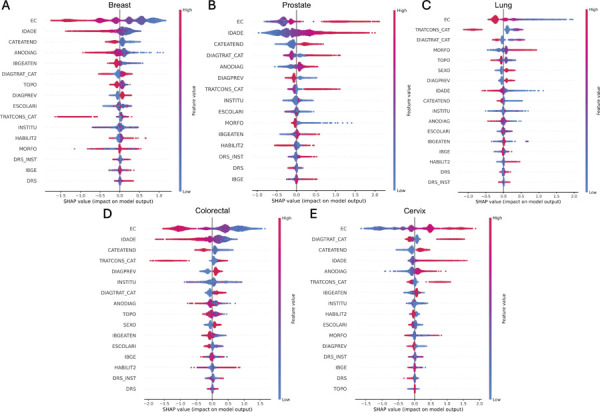
SHAP values for interpretability of the best performing models, according to type of cancer.

ANODIAG: year of diagnosis; CATEATEND: category of care; DIAGPREV: diagnosis prior to admission; DIAGTRAT_CAT: categorized time between diagnosis and start of treatment; DRS: Regional Health Department; DRS_INST: Regional Health Department of the institution; EC: clinical staging; ESCOLARI: schooling; GBS: Gradient Boosting Survival; HABILIT2: high complexity oncology qualification category; IBGE: IBGE code of the city of residence; IBGEATEN: IBGE code of the city of the institution; IDADE: age; INSTITU: institution code; MORFO: tumor morphology; SHAP: SHapley Additive exPlanations; TOPO: tumor topography; TRATCONS_CAT: categorized time between first consultation and start of treatment; XGB-AFT: XGBoost Accelerated Failure Time.

Note: the SHAP values quantify the contribution of each variable to the individual predictions, making it possible to identify both the overall importance of the variables and their impact on the model’s prediction. The beeswarm graphs show the distribution of SHAP values, where each point represents an individual; the color indicates the value of the variable (red = highest values; blue = lowest values), and the position on the x-axis represents the impact of that variable on the prediction. (A) Breast - XGB-AFT; (B) Prostate - GBS; (C) Lung - XGB-AFT; (D) Colorectal - XGB-AFT; (E) Cervix - GBS.

## DISCUSSION

This study stands out by demonstrating the applicability of SML models for predicting survival in patients with five types of cancer. The models performed as assessed by the C-Index, with values between 0.6686 and 0.7845, showing the potential of SML algorithms to predict survival from data collected by cancer registries. The GBS and XGB-AFT models had the best metrics for the various types of cancer. GBS was superior in prostate cancer, while XGB-AFT was the best among the other types of tumors. RSF obtained the third best result for all topographies. It should also be noted that the best algorithms had good predictive performance, above 0.73, regardless of the proportion of censored cases in the sample and the total number of patients included. GBS showed the best performance in terms of IBS across all tumor categories..

In a previous study, our group had already identified good predictive capacity using data from the cancer registry^6^. Considering five-year survival, an accuracy of 77.9% and an AUC of 0.858 were obtained with XGBoost. However, in that study it was necessary to exclude individuals who did not have a complete follow-up period. Thus, out of a total of 31,916 eligible patients, only 23,338 (73%) could be used in the analysis. In another publication, when comparing these results with the SML algorithms, the results showed that the algorithms for dichotomous outcomes underestimated the survival of patients with colorectal cancer by up to 18% in the first year, demonstrating that the SML models are more suitable for this type of analysis.

Considering the weight of the inputs in the model results, the CE variable was the most important in all the analyses, except in the PI for lung cancer, where it came second. This finding is consistent with that of other authors^18^, and demonstrates the importance of staging at diagnosis as the main prognostic factor for different types of cancer.

In breast cancer, the XGB-AFT model performed best, with a C-Index of 0.7845, surpassing the 0.73 obtained in a study of 36,958 patients diagnosed in the Netherlands between 2005 and 2008^19^. In this study, the XGB outperformed the Cox-PH, RSF and SSVM models, whose C-Indexes ranged from 0.63 to 0.64. In the analysis of the importance of variables by SHAP, age and staging stood out as the main predictors, just as we found in our study.

For prostate cancer, the best result was achieved with the GBS model (C-Index = 0.7574), higher than that found in a study using the Surveillance, Epidemiology, and End Results (SEER) database, which brings together data from 18 population-based cancer registries in the United States^20^. Data from patients diagnosed with prostate cancer between 2000 and 2019, with a positive lymph node and no metastases, was used, with a total of 3,280 patients. In this investigation, a C-Index value of 0.745 was obtained with the Gradient Boosting Survival Analysis (GBSA) technique and with the Extra Survival Trees (EST), higher than the other models analyzed, RSF and Cox-PH, which ranged between 0.734 and 0.743.

In the case of lung cancer, the XGB-AFT also stood out (C-Index = 0.7368), surpassing the results obtained in a study using the population-based cancer registry of a state in Germany^21^. Data from patients diagnosed with lung cancer between 2016 and 2021 was used, with a total of 10,383 patients. In this literature study, the RSF model with variable imputation obtained a C-Index of 0.703, while the other approaches (Cox-PH, DeepSurv, and TabNet) performed less well (0.556 to 0.701).

For colorectal cancer, XGB-AFT obtained the highest C-Index in the analysis (0.7618), although this was lower than that found in a study using a hospital database in China^22^. Data from patients diagnosed with colorectal cancer between 2012 and 2019 was used, with a total of 2,157 patients. In the aforementioned study, a C-Index value of 0.789 was obtained for DeepHit, while the other techniques analyzed (Cox-PH, RSF, GB, DeepSurv, Cox-Time, and Neural Multitask Logistic Regression) varied between 0.781 and 0.787. The most important variable in the SHAP analysis of the DeepHit model was staging. It is worth noting that a key variable in the study, the surgical resection margin, is not available in the RHC/SP.

Finally, in cervical cancer, the best algorithm was XGB-AFT, which had a C-Index of 0.7726, lower than the value found in a study using the SEER database^23^. Data from patients diagnosed with cervical cancer between 2013 and 2015 was used, with a total of 3,810 patients. In this literature study, a C-Index value of 0.95 was obtained for the RSF, while the Cox-PH and Weibull obtained 0.81 and 0.80, respectively. The most important variable was T stage, followed by tumor size and staging. Variables related to treatment were used, such as chemotherapy and radiotherapy, in addition to the measurement of tumor size, which are variables we chose not to use in our study.

The use of variables restricted to individual information, such as place of residence, institution of care, clinical characteristics, and time until treatment, without including type of treatment or tumor recurrence, is a strength of the study. This choice broadens the applicability of the models in real planning contexts, as this data is available early in the records. Thus, the algorithms developed can support strategic health management decisions, even in scenarios with limited clinical data. However, the absence of more specific clinical information, such as tumor markers and recurrence data, restricts its use for individual clinical prediction, placing its main contribution in supporting health management and scenario building.

This study has some limitations. Firstly, it is important to note that the study used information from the RHC/SP database. This data is collected in hospitals from medical records by professionals from the institutions themselves, with heterogeneous teams. Although all the registrars receive specific training and use the SISRHC software, which has internal checks and consistency rules capable of standardizing the information, such as restrictions on the selection of morphologies compatible with each topography, inaccuracies can still occur, especially in more complex variables. One attempt to mitigate this risk was to exclude patients without staging or with undefined staging, which suggests poorer quality of the record.

In addition, the database currently does not capture some factors relevant to survival, such as race/skin color, risk factors such as smoking and clinical data such as tumor markers. The latter are particularly important, as they have an impact on more precise definitions of staging and the probability of survival. Therefore, updating registration systems to include new variables could increase the predictive potential of the models.

Additionally, it should be borne in mind that no direct comparison of performance was made with Cox models. The application of this type of model requires the proportionality of risks over time, which may make it unfeasible to include variables that do not meet this premise^2^. Thus, its use could restrict the set of predictor variables considered in the analysis.

Another relevant limitation is that it was not possible to calculate the IBS for the XGB-AFT, XGB-COX, and LGBM models, due to the lack of survival curve estimates with adequate adherence to the observed data, even after adjustment attempts. Future research could explore variations in parameterization, pre-processing or specific methodological adaptations for these algorithms, to make it possible to obtain reliable curves and, consequently, calculate the IBS. It is also worth noting that it was not possible to use SurvSHAP^24^, a specific method for assessing the impact of different variables on survival model predictions. This was because its application requires the generation of individual explanations over multiple survival time points, making processing particularly onerous on extensive bases and in more complex models. We therefore opted to use the traditional SHAP^16^ and PI^17^ for the global assessment of the importance of the variables.

Finally, although the models performed well on RHC/SP data, their application to records from other regions of the country has not been tested. It should be noted that one variable of great importance to the models, clinical staging, has high non-completion values in some regions of the country, which may limit its application. This lack of external validation limits the extrapolation of the results to other states, which could be the subject of future studies exploring the robustness of these models in more heterogeneous national cancer registry databases.

## CONCLUSIONS

The SML algorithms proved to be applicable to the RHC/SP data for survival analysis. GBS, XGB-AFT, and RSF performed best, regardless of sample size or censoring proportion. The results reinforce the potential of these models to support evidence-based public policies, identifying profiles of patients or services at greater risk and contributing to better targeting of resources and organization of care networks.

## Supplementary material

available from: https://doi.org/10.5281/zenodo.19250128

## Data Availability

The data used in this study come from the Registro Hospitalar de Câncer do Estado de São Paulo (São Paulo State Cancer Registry Hospital) and can be accessed at by registering at: https://fosp.saude.sp.gov.br/fosp/diretoria-adjunta-de-informacao-e-epidemiologia/rhc-registro-hospitalar-de-cancer/banco-de-dados-do-rhc/.

## Footnotes

a Supplementary material available from: https://doi.org/10.5281/zenodo.19250128
