# Supplementary material for: Machine learning and cancer survival: analysis of the Registro Hospitalar de Câncer do Estado de São Paulo
Source: Rev Saude Publica. 2026 Jul 24;60:e31. doi: 10.11606/s1518-8787.2026060007389 (PMC13398606; doi:10.11606/s1518-8787.2026060007389)
Supplement: Translation

# Translation: Machine learning e sobrevida em câncer: análise do Registro Hospitalar de Câncer de São Paulo

**Authors:** Fernando Henrique de Albuquerque Maia, Lucas Buk Cardoso, Simone Aldrey Angelo, Yasmin Pacheco Gil Bonilha, Adeylson Guimarães Ribeiro, Maria Paula Curado, Gisele Aparecida Fernandes, Alexandre Dias Porto Chiavegatto, Vanderlei Cunha Parro, Tatiana Natasha Toporcov

**Affiliations:** I Universidade de São Paulo. Faculdade de Saúde Pública. Departamento de Epidemiologia. São Paulo, SP, Brasil; II Instituto Mauá de Tecnologia. Núcleo de Sistemas Eletrônicos Embarcados. São Caetano do Sul, SP, Brasil; III Fundação Oncocentro de São Paulo. São Paulo, SP, Brasil; IV A.C. Camargo Cancer Center. Grupo de Epidemiologia e Estatística em Câncer. São Paulo, SP, Brasil

**Keywords:** Neoplasias, Análise de Sobrevida, Aprendizado de Máquina

## Abstract

**OBJETIVO:**

Comparar o desempenho de diferentes algoritmos de *Survival Machine Learning* (SML) na predição da sobrevida de pacientes com câncer.

**MÉTODOS:**

Utilizaram-se dados do Registro Hospitalar de Câncer do Estado de São Paulo, contemplando os cinco tipos de câncer mais incidentes (mama, próstata, pulmão, colorretal e colo do útero). Foram avaliados seis algoritmos: *Gradient Boosting Survival* (GBS), *Random Survival Forest* (RSF), *Support Vector Machine Survival* (SVM-Survival), *XGBoost Cox*, *XGBoost Accelerated Failure Time* (AFT) e *Light*GBM. O desempenho foi medido pelas métricas *Concordance Index* (C-Index), *C-Index* IPCW e *Integrated Brier Score* (IBS).

**RESULTADOS:**

O modelo *XGBoost* AFT apresentou os melhores resultados de C-Index para mama (0,7845), pulmão (0,7368), colorretal (0,7618) e colo do útero (0,7726), enquanto o GBS foi superior para próstata (0,7574). O estadiamento clínico foi consistentemente a variável mais importante, segundo a análise de explicabilidade.

**CONCLUSÃO:**

Os algoritmos de SML demonstraram bom desempenho preditivo, independentemente do tipo de câncer, do tamanho amostral e da proporção de censura. Esses modelos mostram potencial para subsidiar o planejamento oncológico e apoiar decisões estratégicas na organização das redes de atenção ao câncer.

## INTRODUÇÃO

A incidência de câncer tem aumentado acentuadamente ao longo dos últimos anos, devido ao envelhecimento da população e às mudanças de hábitos de vida, sendo estimados 20 milhões de casos novos e 9,7 milhões de óbitos por ano^1^. É um importante problema de saúde pública, com impacto nos sistemas de saúde em todo o mundo.

A análise de sobrevida é um tipo de análise em que a variável dependente de interesse é o tempo até que ocorra determinado evento^2^. Em câncer, esses estudos visam analisar o tempo entre o diagnóstico e/ou admissão em um serviço especializado no tratamento da doença e o óbito do paciente^3^. Um aspecto importante deste tipo de análise é a inclusão do tempo de observação dos indivíduos censurados. A censura ocorre quando existe perda de seguimento do paciente ou quando o estudo termina e ele segue sem ter apresentado o desfecho de interesse. Em contraposição, análises de desfechos dicotômicos, como sobrevida em cinco anos, precisam utilizar somente os indivíduos sobre os quais há certeza acerca da sobrevida ou do óbito durante o período de acompanhamento, sendo necessário excluir da análise os censurados.

Os modelos de análise de sobrevida baseados em estatística tradicional dependem de premissas acerca da distribuição dos dados. O modelo de riscos proporcionais de Cox (Cox-PH) é o mais utilizado e é um modelo semiparamétrico que assume a proporcionalidade dos riscos para os diferentes indivíduos de maneira constante ao longo do tempo^3^. Desta forma, o uso dele exige a realização de testes que confirmem que as premissas são atendidas, necessitando de adaptações e ajustes quando isso não ocorre^2^. Este modelo é frequentemente utilizado para estimar o efeito de variáveis explicativas sobre o risco de um evento ao longo do tempo, mas pode ser adaptado para predição individual de risco a partir de estimadores como o de Breslow.

O uso dos algoritmos de *Survival Machine Learning* (SML) para análise de sobrevida tem sido crescente nos últimos anos^4,5^. A sua principal vantagem consiste na capacidade de análise de amplos conjuntos de dados com variáveis de diversos formatos, sem necessidade de cumprir determinadas premissas. Isso é especialmente útil em casos de atributos com associações não lineares com a variável de interesse.

No Brasil, alguns trabalhos já abordaram a mortalidade e sobrevida por câncer^6,7^, considerando os dados do Registro Hospitalar de Câncer do Estado de São Paulo (RHC/SP) da Fundação Oncocentro do Estado de São Paulo (FOSP). Entretanto, estes trabalhos não consideraram o tempo de sobrevida enquanto variável dependente de interesse, e sim a ocorrência ou não do evento (óbito) após determinados intervalos de tempo. Desta forma, nestas análises foi necessário excluir indivíduos por perda de seguimento.

Em um estudo prévio, foi realizada a comparação de modelos de SML com modelos de *machine learning* para desfechos dicotômicos de sobrevida em um, três e cinco anos de pacientes com câncer colorretal, demonstrando que os modelos dicotômicos subestimaram a sobrevida destes pacientes^8^. Neste artigo, foram incluídos pacientes com os cinco tipos de câncer mais frequentes no estado de São Paulo, que apresentam diferenças expressivas na sua incidência e na proporção de casos censurados. Foram testados os principais modelos de SML visando identificar os algoritmos que apresentam melhor desempenho na predição e possíveis diferenças de desempenho relacionadas ao tamanho das amostras e à proporção de casos censurados.

## MÉTODOS

O RHC/SP, gerido pela FOSP, reúne informações socioeconômicas e clínicas de pacientes diagnosticados com câncer desde o ano 2000, fornecidas por 81 instituições do estado de São Paulo. Este banco de dados, com informações anonimizadas, é público e pode ser consultado mediante o preenchimento de um cadastro no site da instituição^9^. Para este estudo, foram selecionados casos de cinco tipos de câncer mais incidentes no estado de São Paulo: mama, próstata, pulmão, colorretal e colo do útero.

Previamente à realização do estudo, os autores identificaram as variáveis que seriam incluídas no modelo e os critérios de inclusão e exclusão, considerando as características dos tumores e do conjunto de dados disponível. Optou-se por excluir variáveis que apresentavam alta colinearidade com as variáveis escolhidas, baixa proporção de preenchimento ou que estavam relacionadas ao tipo de tratamento a que o indivíduo foi submetido. A intenção foi trabalhar com dados que estão disponíveis antes de iniciar o tratamento, para possibilitar o uso destes modelos em estudos posteriores com construção de cenários e/ou otimização da organização da rede de serviços de saúde.

A preparação dos conjuntos de dados para os cinco tipos de câncer em estudo ocorreu em duas etapas: seleções gerais e ajustes das colunas – aplicadas a todas as topografias analisadas – e seleções específicas, realizadas de acordo com as particularidades de cada tipo. Para todos os tipos de câncer, foram excluídos pacientes com menos de 20 anos, não residentes no estado de São Paulo, com estadiamento clínico indefinido ou não realizado, com carcinoma *in situ*, sem confirmação microscópica do diagnóstico, com morfologia incerta, ou que tenham realizado transplante de medula óssea.

Nas seleções específicas, foram aplicados critérios adicionais para cada tipo de câncer. Pacientes com câncer de pulmão que receberam hormonioterapia foram excluídos, tendo em vista que este tratamento em geral é destinado a pacientes com outros tipos de tumores com metástases pulmonares. No caso do câncer colorretal, foram considerados apenas os pacientes com morfologia de adenocarcinoma (código 81403), tendo em vista que as outras morfologias são mais raras e apresentam comportamento distinto. Para o câncer de mama, foram incluídas apenas mulheres, tendo em vista que este tipo de câncer em homens é raro e se comporta de forma muito distinta.

O conjunto de variáveis analisado incluiu informações individuais do paciente, como idade (IDADE), sexo (SEXO) e escolaridade (ESCOLARI); variáveis relacionadas ao local de residência, como o código IBGE da cidade (IBGE) e o Departamento Regional de Saúde (DRS); e variáveis referentes à instituição de atendimento, incluindo código da instituição (INSTITU), categoria de atendimento (CATEATEND), código IBGE da cidade da instituição (IBGEATEN), DRS da instituição (DRS_INST) e categoria de habilitação em alta complexidade em oncologia (HABILIT2). Também foram consideradas variáveis clínicas, como diagnóstico prévio à admissão (DIAGPREV), topografia (TOPO), morfologia (MORFO), estadiamento clínico (EC) e ano de diagnóstico (ANODIAG). Por fim, avaliou-se o tempo categorizado entre a primeira consulta e o início do tratamento (TRATCONS_CAT) e entre o diagnóstico e o início do tratamento (DIAGTRAT_CAT). A variável sexo foi utilizada apenas para câncer de pulmão e colorretal, a variável topografia não foi considerada para próstata e a variável morfologia não foi considerada para colorretal. Os dados foram divididos em duas partes, com 80% dos pacientes sendo utilizados para o treinamento dos algoritmos de sobrevida e os outros 20% para validação destes modelos.

Foi considerado como desfecho o tempo entre o diagnóstico e a ocorrência do óbito por todas as causas, sendo incluídos os pacientes censurados. Foram utilizados seis modelos de aprendizado de máquina voltados para a análise de sobrevida: *Gradient Boosting Survival* (GBS), *Random Survival Forest* (RSF), *Survival Support Vector Machine* (SSVM), XG*Boost* Cox (XGB-Cox), XG*Boos*t *Accelerated Failure Time* (XGB-AFT) e *Light*GBM (LGBM). Cada um adota uma estratégia distinta para modelar o tempo até a ocorrência de um evento e foram escolhidos por sua capacidade de lidar com censura nos dados, capturar padrões não lineares e fornecer predições mais flexíveis em comparação com abordagens estatísticas tradicionais. Os aspectos metodológicos relativos ao tratamento do banco de dados, bem como referente às especificidades de cada tipo de modelo de SML, estão descritos de maneira mais aprofundada em outro trabalho^8^.

Para a avaliação dos modelos de sobrevida, foram utilizadas três métricas: *Concordance Index* (C-Index), *Inverse Probability of Censoring Weighted Concordance Index* (C-Index IPCW) *e Integrated Brier Score* (IBS). O C-Index é uma métrica empregada para medir a capacidade discriminatória do modelo, ou seja, sua habilidade de ordenar corretamente os tempos de sobrevida dos indivíduos^10^. Valores próximos a 1 indicam concordância perfeita, enquanto um valor de 0,5 sugere desempenho equivalente ao acaso. O C-Index IPCW é uma variação do C-Index que corrige vieses introduzidos pela censura não informativa ou dependente de covariáveis^11^. Por fim, o IBS mede a exatidão das predições de probabilidade de sobrevivência ao longo do tempo, sendo calculado a partir das curvas de sobrevida estimadas pelos modelos. Valores mais baixos indicam melhor desempenho, sendo 0 a predição perfeita^12^. Como o IBS depende das curvas de sobrevida, ele somente foi calculado para os modelos que apresentaram curvas de sobrevida adequadas e consistentes com as observações empíricas.

A seleção dos hiperparâmetros dos modelos de aprendizado de máquina foi realizada com o Optuna^13^, utilizando três amostradores distintos. O RandomSampler combina os parâmetros de forma aleatória; o TPESampler^14^ otimiza a escolha por meio de modelos probabilísticos; e o CmaEsSampler^15^ emprega uma estratégia evolucionária que ajusta uma distribuição gaussiana sobre o espaço de hiperparâmetros. Cada amostrador foi avaliado com 150 combinações distintas, aplicando validação cruzada em 10 *folds* para cada conjunto de parâmetros. Para cada tipo de câncer, selecionou-se o modelo com melhor desempenho entre os três amostradores.

Para avaliar o impacto das diferentes variáveis nas predições dos modelos de sobrevida, utilizou-se o *SHapley Additive exPlanations* (SHAP)^16^ e o *Permutation Importance* (PI)^17^. O SHAP estima a contribuição média de cada variável para as predições, considerando todas as combinações possíveis de valores, enquanto o PI avalia a perda de desempenho do modelo quando os valores de uma variável são embaralhados de maneira aleatória.

### Aspectos Éticos

Este estudo foi realizado com uma base de dados pública, anonimizada e sem variáveis sensíveis que permitissem identificação dos indivíduos, preservando a confidencialidade. Desta forma, não foi submetido a um Comitê de Ética em Pesquisa, de acordo com a Resolução nº 510/2016 do Conselho Nacional de Saúde.

## RESULTADOS

O banco de dados foi obtido do RHC/SP em setembro de 2024 com dados de 1.223.973 indivíduos. Após as seleções gerais e específicas por tipo de câncer, foram incluídos 141.726 pacientes com câncer de mama, 111.406 com câncer de próstata, 45.719 com câncer de pulmão, 44.856 com câncer colorretal e 27.850 com câncer do colo do útero, conforme descrito no Quadro 1.

**Quadro 1:** Ajustes e seleções feitas nos conjuntos de dados do RHC/SP.

	Total de pacientes no RHC/SP	Exclusões gerais	Seleções específicas	Total de pacientes incluídos
Câncer de mama (C50)	170.413	Idade < 20 anos Residentes em outros estados Estadiamento clínico indefinido ou não realizado Sem confirmação microscópica do diagnóstico Morfologia incerta Carcinoma *in situ* Realização de transplante de medula óssea	Sexo feminino	141.726
Câncer de próstata (C61)	128.575		(Nenhuma seleção específica adicional)	111.406
Câncer de pulmão (C34)	57.620		Sem tratamento com hormonioterapia	45.719
Câncer colorretal (C18, C19, C20)	90.625		Adenocarcinoma (morfologia = 81403)	44.856
Câncer do colo do útero (C53)	56.912		(Nenhuma seleção específica adicional)	27.850

RHC/SP: Registro Hospitalar de Câncer do estado de São Paulo.

O perfil dos pacientes incluídos no estudo é apresentado na Tabela 1. O número de pacientes incluídos para cada tipo de câncer variou de 27.850 casos de colo do útero a 141.726 casos de mama, passando por 44.856 de colorretal, 45.719 de pulmão e 111.406 de próstata. No câncer de pulmão e no câncer colorretal, observou-se predomínio de pacientes do sexo masculino, correspondendo a 60,2% e 52,0% dos casos, respectivamente.

**Tabela 1:** Perfil de pacientes incluídos no estudo.

	Mama n (%)	Próstata n (%)	Pulmão n (%)	Colorretal n (%)	Colo do útero n (%)
Escolaridade					
Analfabeto	4.997 (3,53)	5.257 (4,72)	2.418 (5,29)	1.670 (3,72)	2.066 (7,42)
Ensino Fundamental Incompleto	33.601 (23,71)	34.875 (31,30)	13.717 (30,00)	11.549 (25,75)	8.167 (29,33)
Ensino Fundamental Completo	22.536 (15,90)	18.557 (16,66)	8.967 (19,61)	8.198 (18,28)	4.527 (16,26)
Ensino Médio	25.771 (18,18)	13.009 (11,68)	5.387 (11,78)	6.302 (14,05)	4.022 (14,44)
Ensino Superior	15.651 (11,04)	7.986 (7,17)	3.022 (6,61)	3.431 (7,65)	1.197 (4,30)
Sem informação	39.170 (27,64)	31.722 (28,47)	12.208 (26,70)	13.706 (30,56)	7.871 (28,26)
Idade - Média (Mediana)	56,46 (56)	67,58 (68)	64,01 (64)	62,39 (63)	51,53 (51)
Sexo					
Masculino	0 (0)	111.406 (100)	27.530 (60,21)	23.312 (51,97)	0 (0)
Feminino	141.726 (100)	0 (0)	18.189 (39,78)	21.544 (48,03)	27.850 (100)
Categoria de atendimento					
Convênio	16.487 (11,63)	10.621 (9,53)	3.544 (7,75)	3.991 (8,90)	1.064 (3,82)
SUS	82.721 (58,37)	68.698 (61,66)	25.477 (55,72)	28.149 (62,75)	16.426 (58,98)
Particular	906 (0,64)	1.691 (1,52)	341 (0,74)	299 (0,67)	74 (0,27)
Sem Informação	41.612 (29,36)	30.396 (27,28)	16.357 (35,77)	12.417 (27,68)	10.286 (36,93)
Diagnóstico prévio					
Sem diagnóstico prévio	69.411 (48,98)	36.225 (32,52)	29.816 (65,22)	18.255 (40,70)	11.761 (42,23)
Com diagnóstico prévio	72.315 (51,02)	75.181 (67,48)	15.903 (34,78)	26.601 (59,30)	16.089 (57,77)
Topografia	C500: 4.512 (3,18) C501: 6.651 (4,69) C502: 9.188 (6,48) C503: 5.195 (3,67) C504: 34.244 (24,16) C505: 6.339 (4,47) C506: 1.300 (0,92) C508: 21.803 (15,38) C509: 52.494 (37,04)	C619: 111.406 (100)	C340: 2.499 (5,47) C341: 12.982 (28,40) C342: 1.340 (2,93) C343: 6.736 (14,73) C348: 1.679 (3,67) C349: 20.483 (44,80)	C180: 1.371 (3,06) C181: 81 (0,18) C182: 3.547 (7,91) C183: 379 (0,84) C184: 1.616 (3,60) C185: 327 (0,73) C186: 1.996 (4,45) C187: 6.638 (14,80) C188: 297 (0,66) C189: 6.955 (15,51) C199: 2.791 (6,22) C209: 18.858 (42,04)	C530: 759 (2,73) C531: 1.243 (4,46) C538: 443 (1,59) C539: 25.405 (91,22)
Estadiamento clínico					
I	33.780 (23,83)	9.133 (8,20)	4.936 (10,80)	5.378 (11,99)	8.346 (29,97)
II	54.970 (38,79)	65.986 (59,23)	2.937 (6,42)	12.400 (27,64)	6.544 (23,50)
III	38.224 (26,97)	18.659 (16,75)	12.037 (26,33)	14.393 (32,09)	8.722 (31,32)
IV	14.752 (10,41)	17.628 (15,82)	25.809 (56,45)	12.685 (28,28)	4.238 (15,22)
Tempo categorizado entre a primeira consulta e o início do tratamento					
Até 60 dias	84.201 (59,41)	51.357 (46,10)	25.728 (56,27)	31.905 (71,13)	15.504 (55,67)
Entre 61 e 90 dias	23.604 (16,65)	13.299 (11,94)	5.056 (11,06)	5.030 (11,21)	4.709 (16,91)
Mais de 91 dias	31.390 (22,15)	39.839 (35,76)	7.008 (15,33)	5.743 (12,80)	6.245 (22,42)
Não tratou	2.531 (1,79)	6.911 (6,20)	7.927 (17,34)	2.178 (4,86)	1.392 (5,00)
Tempo categorizado entre o diagnóstico e o início do tratamento					
Até 60 dias	69.639 (49,14)	31.301 (28,10)	26.809 (58,64)	25.554 (56,97)	11.608 (41,68)
Entre 61 e 90 dias	26.803 (18,91)	14.725 (13,22)	5.035 (11,01)	7.019 (15,65)	5.211 (18,71)
Mais de 91 dias	42.753 (30,17)	58.469 (52,48)	5.948 (13,01)	10.105 (22,53)	9.639 (34,61)
Não tratou	2.531 (1,79)	6.911 (6,20)	7.927 (17,34)	2.178 (4,86)	1.392 (5,00)
Óbito por todas as causas					
Não	95.069 (67,08)	75.027 (67,35)	6.808 (14,89)	22.979 (51,23)	15.333 (55,06)
Sim	46.657 (32,92)	36.379 (32,65)	38.911 (85,11)	21.877 (48,77)	12.517 (44,94)
Divisão do modelo					
Treino	113.380 (80,00)	89.124 (80,00)	36.575 (80,00)	35.884 (80,00)	22.280 (80,00)
Teste	28.346 (20,00)	22.282 (20,00)	9.144 (20,00)	8.972 (20,00)	5.570 (20,00)
Total	141.726 (100)	111.406 (100)	45.719 (100)	44.856 (100)	27.850 (100)

A proporção de óbitos por todas as causas variou consideravelmente entre os tipos de câncer incluídos. Os casos de câncer de pulmão apresentaram a maior proporção de óbitos (85,1%), enquanto mama e próstata registraram a menor proporção de óbitos com valores próximos (32,9% e 32,7%, respectivamente). Colorretal (48,8%) e colo do útero (44,9%) situaram-se em patamares intermediários. A distribuição de acordo com a topografia, bem como a divisão entre treino e teste encontra-se no Material Suplementar^a^. As curvas de Kaplan-Meier estratificadas por estadiamento clínico também se encontram no Material Suplementar.

A Tabela 2 apresenta o desempenho dos modelos de SML para predição de sobrevida após a otimização dos hiperparâmetros para os cinco tipos de câncer. No câncer de mama, o modelo XGB-AFT apresentou o melhor desempenho em termos de C-Index (0,7845) e C-Index IPCW (0,7570), enquanto o GBS obteve o menor IBS (0,1325). Já no câncer de próstata, o GBS destacou-se com os melhores valores de C-Index (0,7574) e C-Index IPCW (0,7332), além do menor IBS (0,1298). No cenário do câncer de pulmão, o modelo XGB-AFT obteve o maior C-Index (0,7368) e o maior C-Index IPCW (0,7325), embora o menor IBS tenha sido registrado pelo modelo GBS (0,1165). Para o câncer colorretal, o XGB-AFT apresentou novamente os maiores valores de C-Index (0,7618) e C-Index IPCW (0,7532), enquanto o menor IBS foi registrado pelo GBS (0,1552). Finalmente, no caso do câncer do colo do útero, o modelo XGB-Cox alcançou o maior C-Index (0,7726), enquanto o GBS obteve o maior C-Index IPCW (0,7643) e o menor IBS (0,1473). As curvas de sobrevida estão apresentadas no Material Suplementar.

**Tabela 2:** Sumário dos resultados obtidos pelos melhores modelos após a busca pelos hiperparâmetros.

Modelos	Câncer de mama			Câncer de próstata			Câncer de pulmão			Câncer colorretal			Câncer do colo do útero		
	C-Index	C-Index IPCW	IBS	C-Index	C-Index IPCW	IBS	C-Index	C-Index IPCW	IBS	C-Index	C-Index IPCW	IBS	C-Index	C-Index IPCW	IBS
RSF	0,7784	0,7525	0,1335	0,7557	0,7300	0,1303	0,7247	0,7212	0,1192	0,7547	0,7489	0,1565	0,7664	0,7583	0,1481
GBS	0,7821	0,7565	**0,1324**	**0,7574**	**0,7332**	**0,1298**	0,7324	0,7288	**0,1165**	0,7588	0,7526	**0,1552**	0,7722	**0,7643**	**0,1473**
SSVM	0,7574	0,7301	0,1415	0,7094	0,6907	0,1424	0,6686	0,6669	0,1310	0,7088	0,7036	0,1774	0,7372	0,7315	0,1630
XGB - Cox	0,7021	0,6896	-	0,6747	0,6645	-	0,7212	0,7174	-	0,7240	0,7184	-	0,7452	0,7375	-
XGB - AFT	**0,7845**	**0,7570**	-	0,7574	0,7328	-	**0,7368**	**0,7325**	-	**0,7618**	**0,7532**	-	**0,7726**	0,7639	-
LGBM	0,7133	0,7000	-	0,6862	0,6742	-	0,7210	0,7170	-	0,7275	0,7211	-	0,7483	0,7404	-

Nota: em negrito, destacam-se os modelos que apresentaram o melhor desempenho para cada tipo de tumor e métrica avaliada.

C-Index: *Concordance Index*; C-Index IPCW: *Inverse Probability of Censoring Weighted Concordance Index*; GBS: *Gradient Boosting Survival*; IBS: *Integrated Brier Score*; LGBM: *LightGBM;* RSF: *Random Survival Forest*; SSVM: *Survival Support Vector Machine*; XGB-Cox: *XGBoost Cox*; XGB-AFT: *XGBoost Accelerated Failure Time.*

O Quadro 2 apresenta as variáveis mais relevantes para a predição de sobrevida, identificadas por meio dos métodos SHAP e PI, para cada tipo de câncer analisado. Os modelos selecionados foram aqueles que apresentaram melhor desempenho na Tabela 2 de acordo com o C-Index IPCW: XGB-AFT para mama, pulmão e colorretal; GBS para próstata e colo do útero. A variável EC (estadiamento clínico) foi de forma consistente classificada como a mais importante nos modelos de câncer de mama, próstata, colo do útero e colorretal, tanto pelos métodos SHAP quanto PI, enquanto no câncer de pulmão ela foi a mais importante apenas de acordo com o método SHAP, sendo que no método PI o tempo categorizado entre a consulta e o tratamento foi a variável mais importante. A Figura apresenta o gráfico com a distribuição dos valores de SHAP.

**Quadro 2:** Importância das *features* pelos métodos SHAP e *Permutation Importance*.

	Câncer de mama		Câncer de próstata		Câncer de pulmão		Câncer colorretal		Câncer do colo do útero	
	XGB - AFT		GBS		XGB - AFT		XGB - AFT		GBS	
	SHAP	PI	SHAP	PI	SHAP	PI	SHAP	PI	SHAP	PI
1	EC	EC	EC	EC	EC	TRATCONS_CAT	EC	EC	EC	EC
2	IDADE	IDADE	IDADE	IDADE	TRATCONS_CAT	EC	IDADE	TRATCONS_CAT	DIAGTRAT_CAT	CATEATEND
3	CATEATEND	CATEATEND	CATEATEND	CATEATEND	DIAGTRAT_CAT	DIATRAT_CAT	CATEATEND	IDADE	CATEATEND	DIAGTRAT_CAT
4	ANODIAG	ANODIAG	DIAGTRAT_CAT	DIAGTRAT_CAT	MORFO	MORFO	TRATCONS_CAT	INSTITU	IDADE	IDADE
5	IBGEATEN	IBGEATEN	ANODIAG	ANODIAG	TOPO	INSTITU	DIAGPREV	CATEATEND	ANODIAG	ANODIAG
6	DIAGTRAT_CAT	INSTITU	DIAGPREV	INSTITU	SEXO	TOPO	INSTITU	ANODIAG	TRATCONS_CAT	INSTITU
7	TOPO	TRATCONS_CAT	TRATCONS_CAT	TRATCONS_CAT	DIAGPREV	DIAGPREV	DIAGTRAT_CAT	DIAGTRAT_CAT	IBGEATEN	TRATCONS_CAT
8	DIAGPREV	ESCOLARI	INSTITU	IBGEATEN	IDADE	IDADE	ANODIAG	TOPO	INSTITU	IBGEATEN
9	ESCOLARI	DIAGTRAT_CAT	MORFO	DRS_INST	CATEATEND	CATEATEND	TOPO	IBGEATEN	HABILIT2	ESCOLARI
10	TRATCONS_CAT	TOPO	ESCOLARI	HABILIT2	INSTITU	ANODIAG	SEXO	DIAGPREV	ESCOLARI	MORFO

ANODIAG: ano do diagnóstico; CATEATEND: categoria de atendimento; DIAGPREV: diagnóstico prévio à admissão; DIAGTRAT_CAT: tempo categorizado entre diagnóstico e início do tratamento; DRS: Departamento Regional de Saúde; DRS_INST: Departamento Regional de Saúde da instituição; EC: estadiamento clínico; ESCOLARI: escolaridade; GBS: *Gradient Boosting Survival*; HABILIT2: categoria de habilitação em alta complexidade em oncologia; IBGE: código IBGE da cidade de residência; IBGEATEN: código IBGE da cidade da instituição; IDADE: idade; INSTITU: código da instituição; MORFO: morfologia tumoral; PI: *Permutation Importance*; SHAP: *SHapley Additive exPlanations*; TOPO: topografia tumoral; TRATCONS_CAT: tempo categorizado entre primeira consulta e início do tratamento; XGB-AFT: *XGBoost Accelerated Failure Time*.

**Figura:** Valores de SHAP para interpretabilidade dos modelos de melhor desempenho, segundo tipo de câncer.

ANODIAG: ano do diagnóstico; CATEATEND: categoria de atendimento; DIAGPREV: diagnóstico prévio à admissão; DIAGTRAT_CAT: tempo categorizado entre diagnóstico e início do tratamento; DRS: Departamento Regional de Saúde; DRS_INST: Departamento Regional de Saúde da instituição; EC: estadiamento clínico; ESCOLARI: escolaridade; GBS: *Gradient Boosting Survival*; HABILIT2: categoria de habilitação em alta complexidade em oncologia; IBGE: código IBGE da cidade de residência; IBGEATEN: código IBGE da cidade da instituição; IDADE: idade; INSTITU: código da instituição; MORFO: morfologia tumoral; PI: *Permutation Importance*; SHAP: *SHapley Additive exPlanations*; TOPO: topografia tumoral; TRATCONS_CAT: tempo categorizado entre primeira consulta e início do tratamento; XGB-AFT: *XGBoost Accelerated Failure Time*.

Nota: os valores de SHAP quantificam a contribuição de cada variável para as predições individuais, permitindo identificar tanto a importância global das variáveis quanto seu impacto na predição do modelo. Os gráficos do tipo *beeswarm* mostram a distribuição dos valores de SHAP, em que cada ponto representa um indivíduo; a cor indica o valor da variável (vermelho = valores mais altos; azul = valores mais baixos), e a posição no eixo x representa o impacto dessa variável na predição. (A) Mama - XGB-AFT; (B) Próstata - GBS; (C) Pulmão - XGB-AFT; (D) Colorretal - XGB-AFT; (E) Colo do útero - GBS.

## DISCUSSÃO

Este estudo se destaca ao demonstrar a aplicabilidade de modelos de SML para a predição da sobrevida em pacientes com cinco tipos de câncer. Os modelos apresentaram desempenho avaliado pelo C-Index, com valores entre 0,6686 e 0,7845, evidenciando o potencial dos algoritmos de SML para predizer a sobrevida a partir de dados coletados pelos registros de câncer. Os modelos GBS e XGB-AFT foram os que apresentaram as melhores métricas nos diversos tipos de câncer. O GBS foi superior no câncer de próstata, enquanto o XGB-AFT foi o melhor nos demais tipos de tumores. Já o RSF obteve o terceiro melhor resultado para todas as topografias. Ressalta-se ainda que os melhores algoritmos obtiveram bom desempenho preditivo, acima de 0,73, independentemente da proporção de casos censurados na amostra e do número total de pacientes incluídos. O GBS apresentou o melhor desempenho em relação ao IBS em todas as categorias de tumores.

Em estudo prévio, nosso grupo já havia identificado boa capacidade de predição a partir dos dados do registro de câncer^6^. Considerando a sobrevida em cinco anos, obteve-se uma acurácia de 77,9% e uma AUC de 0,858 com o XGBoost. Entretanto, naquele estudo foi necessário excluir os indivíduos que não apresentavam período completo de seguimento. Desta forma, de um total de 31.916 pacientes elegíveis, somente 23.338 (73%) puderam ser utilizados na análise. Em outra publicação, ao realizar a comparação entre estes resultados e os algoritmos de SML, os resultados demonstraram que os algoritmos para desfecho dicotômicos subestimaram a sobrevida de pacientes com câncer colorretal em até 18% no 1º ano, demonstrando que os modelos de SML são mais adequados para este tipo de análise.

Considerando o peso das entradas nos resultados dos modelos, a variável EC foi a mais importante em todas as análises, exceto na PI para o câncer de pulmão, em que ela ficou em segundo lugar. Este achado é consistente com o de outros autores^18^, e demonstra importância do estadiamento ao diagnóstico como principal fator prognóstico dos diversos tipos de câncer.

No câncer de mama, o modelo XGB-AFT apresentou o melhor desempenho, com C-Index de 0,7845, superando os 0,73 obtidos em um estudo com 36.958 pacientes diagnosticadas na Holanda entre 2005 e 2008^19^. No estudo citado, o XGB obteve desempenho superior aos modelos Cox-PH, RSF e SSVM, cujos C-Indexes variaram entre 0,63 e 0,64. Na análise de importância das variáveis por SHAP, idade e estadiamento destacaram-se como os principais preditores, da mesma forma que encontramos no nosso estudo.

Para o câncer de próstata, o melhor resultado foi alcançado com o modelo GBS (C-Index = 0,7574), superior ao encontrado em um estudo que utilizou a base de dados *Surveillance, Epidemiology, and End Results* (SEER), que congrega os dados de 18 registros de câncer de base populacional nos Estados Unidos^20^. Foram utilizados os dados de pacientes diagnosticados com câncer de próstata entre 2000 e 2019, com linfonodo positivo e sem metástases, com um total de 3.280 pacientes. Na referida investigação, foi obtido um valor de C-Index de 0,745 com a técnica *Gradient Boosting Survival Analysis* (GBSA) e com a *Extra Survival Trees* (EST), superior ao dos demais modelos analisados, RSF e Cox-PH, que variaram entre 0,734 e 0,743.

No caso do câncer de pulmão, o XGB-AFT também se destacou (C-Index = 0,7368), superando os resultados obtidos em estudo que utilizou o registro de câncer de base populacional de um estado na Alemanha^21^. Foram utilizados os dados de pacientes diagnosticados com câncer de pulmão entre 2016 e 2021, com um total de 10.383 pacientes. Nesse estudo da literatura, o modelo RSF com imputação de variáveis obteve C-Index de 0,703, enquanto as demais abordagens (Cox-PH, DeepSurv e TabNet) apresentaram desempenho inferior (0,556 a 0,701).

Para o câncer colorretal, o XGB-AFT obteve o maior C-Index da análise (0,7618), embora inferior ao encontrado em estudo desenvolvido com uma base de dados hospitalar na China^22^. Foram utilizados os dados de pacientes diagnosticados com câncer colorretal entre 2012 e 2019, com um total de 2.157 pacientes. No estudo citado, foi obtido o valor de C-Index de 0,789 para o DeepHit, enquanto as demais técnicas analisadas (Cox-PH, RSF, GB, DeepSurv, Cox-Time e *Neural Multitask Logistic Regression*) variaram entre 0,781 e 0,787. A variável mais importante na análise SHAP do modelo DeepHit foi o estadiamento. Cabe destacar que uma variável de destaque no estudo, a margem de ressecção cirúrgica, não está disponível no RHC/SP.

Por fim, no câncer do colo do útero, o melhor algoritmo foi o XGB-AFT, que apresentou C-Index de 0,7726, abaixo do valor encontrado em estudo desenvolvido com a base de dados SEER^23^. Foram utilizados os dados de pacientes diagnosticadas com câncer do colo do útero entre 2013 e 2015, com um total de 3.810 pacientes. Nesse estudo da literatura, foi obtido o valor de C-Index de 0,95 para o RSF, enquanto o Cox-PH e Weibull obtiveram 0,81 e 0,80, respectivamente. A variável mais importante foi estágio T, seguida por tamanho do tumor e estadiamento. Foram utilizadas variáveis relacionadas ao tratamento, como quimioterapia e radioterapia, além da medida do tamanho do tumor, que são variáveis que optamos por não utilizar no nosso estudo.

O uso de variáveis restritas a informações individuais, do local de residência, instituição de atendimento, características clínicas e tempo até o tratamento, sem incluir tipo de tratamento ou recorrência tumoral, constitui uma fortaleza do estudo. Essa escolha amplia a aplicabilidade dos modelos em contextos reais de planejamento, pois tais dados estão disponíveis precocemente nos registros. Assim, os algoritmos desenvolvidos podem apoiar decisões estratégicas da gestão em saúde, mesmo em cenários com dados clínicos limitados. Entretanto, a ausência de informações clínicas mais específicas, como marcadores tumorais e dados de recorrência, restringe seu uso para predição clínica individual, situando sua principal contribuição no apoio à gestão e à construção de cenários em saúde.

Este estudo apresenta algumas limitações. A princípio, é importante destacar que o estudo utilizou informações do banco de dados do RHC/SP. Estes dados são coletados nos hospitais a partir dos prontuários por profissionais das próprias instituições, com equipes heterogêneas na sua composição. Embora todos os registradores recebam treinamento específico e utilizem o *software* SISRHC, que possui verificações internas e regras de consistência capazes de padronizar as informações, como restrição na seleção de morfologias compatíveis com cada topografia, ainda assim, podem ocorrer imprecisões, especialmente em variáveis mais complexas. Uma tentativa de mitigar este risco foi a exclusão de pacientes sem estadiamento ou com estadiamento indefinido, que sugerem pior qualidade do registro.

Além disso, a base não captura atualmente alguns fatores relevantes para a sobrevida, como raça/cor, fatores de risco como tabagismo e dados clínicos como marcadores tumorais. Estes últimos têm especial importância, tendo em vista que impactam em definições mais precisas acerca do estadiamento e da probabilidade de sobrevida. Desta forma, a atualização dos sistemas de registro para inclusão de novas variáveis poderá ampliar o potencial preditivo dos modelos.

Adicionalmente, deve ser considerado que não foi realizada comparação direta de desempenho com modelos de Cox. A aplicação desse tipo de modelo exige a proporcionalidade dos riscos ao longo do tempo, o que pode inviabilizar a inclusão de variáveis que não atendam a essa premissa^2^. Assim, sua utilização poderia restringir o conjunto de variáveis preditoras consideradas na análise.

Outra limitação relevante é que não foi possível calcular o IBS para os modelos XGB-AFT, XGB-COX e LGBM, em razão da ausência de estimativas de curvas de sobrevida com aderência adequada aos dados observados, mesmo após tentativas de ajuste. Futuras investigações poderão explorar variações de parametrização, pré-processamento ou adaptações metodológicas específicas para esses algoritmos, de modo a viabilizar a obtenção de curvas confiáveis e, consequentemente, o cálculo do IBS. Além disso, cabe destacar a impossibilidade de utilizar o SurvSHAP^24^, método específico para avaliar impacto das diferentes variáveis nas predições modelos de sobrevida. Isso ocorreu porque sua aplicação exige a geração de explicações individuais ao longo de múltiplos pontos do tempo de sobrevida, tornando o processamento particularmente oneroso em bases extensas e em modelos mais complexos. Desta forma, optou-se pela adoção do SHAP^16^ tradicional e do PI^17^ para a avaliação global da importância das variáveis.

Por fim, embora os modelos tenham apresentado bom desempenho nos dados do RHC/SP, sua aplicação em registros de outras regiões do país não foi testada. Destaca-se que uma variável de grande importância para os modelos, estadiamento clínico, tem valores de não-preenchimento elevados em algumas regiões do País, o que pode limitar a sua aplicação. Essa ausência de validação externa limita a extrapolação dos resultados para outros estados, o que pode ser objeto de futuros estudos que explorem a robustez desses modelos em bases nacionais de registro de câncer mais heterogêneas.

## CONCLUSÕES

Os algoritmos de SML mostraram-se aplicáveis aos dados do RHC/SP para análise de sobrevida. O GBS, o XGB-AFT e o RSF apresentaram melhor desempenho, independentemente do tamanho da amostra ou da proporção de censura. Os resultados reforçam o potencial desses modelos para apoiar políticas públicas orientadas por evidências, identificando perfis de pacientes ou serviços com maior risco e contribuindo para o melhor direcionamento de recursos e organização das redes assistenciais.

## Material suplementar

disponível em: https://doi.org/10.5281/zenodo.19250128

## Data Availability

Os dados utilizados neste estudo são provenientes do Registro Hospitalar de Câncer do Estado de São Paulo e o acesso a eles pode ser feito pelo site mediante cadastro: https://fosp.saude.sp.gov.br/fosp/diretoria-adjunta-de-informacao-e-epidemiologia/rhc-registro-hospitalar-de-cancer/banco-de-dados-do-rhc/

## Footnotes

a Material suplementar disponível em: https://doi.org/10.5281/zenodo.19250128
